# Adherence to Mediterranean diet and health status: meta-analysis

**DOI:** 10.1136/bmj.a1344

**Published:** 2008-09-11

**Authors:** Francesco Sofi, Francesca Cesari, Rosanna Abbate, Gian Franco Gensini, Alessandro Casini

**Affiliations:** 1 Department of Medical and Surgical Critical Care, Thrombosis Centre, University of Florence, Viale Morgagni 85, 50134 Florence, Italy; 2 Regional Agency for Nutrition, Azienda Ospedaliero-Universitaria Careggi, Florence; 3 Don Carlo Gnocchi Foundation, Onlus IRCCS, Impruneta, Florence; 4 Department of Clinical Pathophysiology, Unit of Clinical Nutrition, University of Florence; 5 Centro Interdipartimentale per la Ricerca e la Valorizzazione degli Alimenti (CeRA), University of Florence

## Abstract

**Objective** To systematically review all the prospective cohort studies that have analysed the relation between adherence to a Mediterranean diet, mortality, and incidence of chronic diseases in a primary prevention setting.

**Design** Meta-analysis of prospective cohort studies.

**Data sources** English and non-English publications in PubMed, Embase, Web of Science, and the Cochrane Central Register of Controlled Trials from 1966 to 30 June 2008.

**Studies reviewed** Studies that analysed prospectively the association between adherence to a Mediterranean diet, mortality, and incidence of diseases; 12 studies, with a total of 1 574 299 subjects followed for a time ranging from three to 18 years were included.

**Results** The cumulative analysis among eight cohorts (514 816 subjects and 33 576 deaths) evaluating overall mortality in relation to adherence to a Mediterranean diet showed that a two point increase in the adherence score was significantly associated with a reduced risk of mortality (pooled relative risk 0.91, 95% confidence interval 0.89 to 0.94). Likewise, the analyses showed a beneficial role for greater adherence to a Mediterranean diet on cardiovascular mortality (pooled relative risk 0.91, 0.87 to 0.95), incidence of or mortality from cancer (0.94, 0.92 to 0.96), and incidence of Parkinson’s disease and Alzheimer’s disease (0.87, 0.80 to 0.96).

**Conclusions** Greater adherence to a Mediterranean diet is associated with a significant improvement in health status, as seen by a significant reduction in overall mortality (9%), mortality from cardiovascular diseases (9%), incidence of or mortality from cancer (6%), and incidence of Parkinson’s disease and Alzheimer’s disease (13%). These results seem to be clinically relevant for public health, in particular for encouraging a Mediterranean-like dietary pattern for primary prevention of major chronic diseases.

## Introduction

The Mediterranean diet, representing the dietary pattern usually consumed among the populations bordering the Mediterranean sea, has been widely reported to be a model of healthy eating for its contribution to a favourable health status and a better quality of life.^1^ ^2^ Since the first data from the seven countries study,^3^ several studies in different populations have established a beneficial role for the main components of the Mediterranean diet on the occurrence of cardiovascular diseases and chronic degenerative diseases.^2^ ^4^ However, research interest in this field over the past years has been focused on estimating adherence to the whole Mediterranean diet rather than analysing the individual components of the dietary pattern in relation to the health status of the population.^5^ This because the analyses of single nutrients ignore important interactions between components of a diet and, more importantly, because people do not eat isolated nutrients. Hence, dietary scores estimating adherence to a Mediterranean diet, devised a priori on the basis of the characteristic components of the traditional diet of the Mediterranean area, have been found to be associated with a reduction of overall mortality and mortality from cardiovascular diseases and cancer.^6^ The aim of this study was to do a systematic review with meta-analysis of all the available prospective cohort studies that have assessed the association between adherence to a Mediterranean diet and adverse outcomes, in order to establish the role of adherence to a Mediterranean diet in primary prevention.

## Methods

### Data sources

We focused on prospective studies investigating the association between adherence to a Mediterranean diet and health outcomes. We searched PubMed, Embase, Web of Science, and the Cochrane Central Register of Controlled Trials databases up to 30 June 2008, using a search strategy that included both truncated free text and exploded MeSH terms. MeSH headings included “Mediterranean”, “diet”, “dietary pattern”, “disease”, “health”, “cardiovascular disease”, “cerebrovascular disease”, “coronary heart disease”, “degenerative diseases”, “cancer”, “neoplasm”, “prospective”, “follow-up”, or “cohort”, and their variants. The search strategy had no language restrictions. We also consulted references from the extracted articles and reviews to complete the data bank. When multiple articles for a single study were present, we used the latest publication and supplemented it, if necessary, with data from the most complete or updated publication. We assessed the relevance of studies by using a hierarchical approach based on title, abstract, and the full manuscript.

### Study selection

We identified studies that prospectively evaluated the association of an a priori score used for assessing adherence to a Mediterranean diet and adverse clinical outcomes. We excluded the studies if they had a cross sectional or case-control design, if they analysed adherence to a non-specific dietary pattern or to a recommended dietary guideline and not to a Mediterranean diet, if they evaluated a cohort of patients with a previous clinical event (that is, secondary prevention), if they did not adjust for potential confounders, and if they did not report an adequate statistical analysis.

Figure 1 shows the process of study selection. Our initial search yielded 62 reports, of which we excluded 20 on the basis of the title or abstract. Of the remaining 42 articles, we excluded 26 for the following reasons: a non-specific dietary pattern, instead of a Mediterranean diet, was evaluated (n=3); cross sectional or case-control design was used (n=18); and the study population was in secondary prevention (n=5). We excluded four additional articles because they represented duplicate studies, so we included only the latest or the more complete paper in the final analysis. Finally, 12 articles fulfilled our inclusion criteria.^w1-w12^

**Figure fig1:**
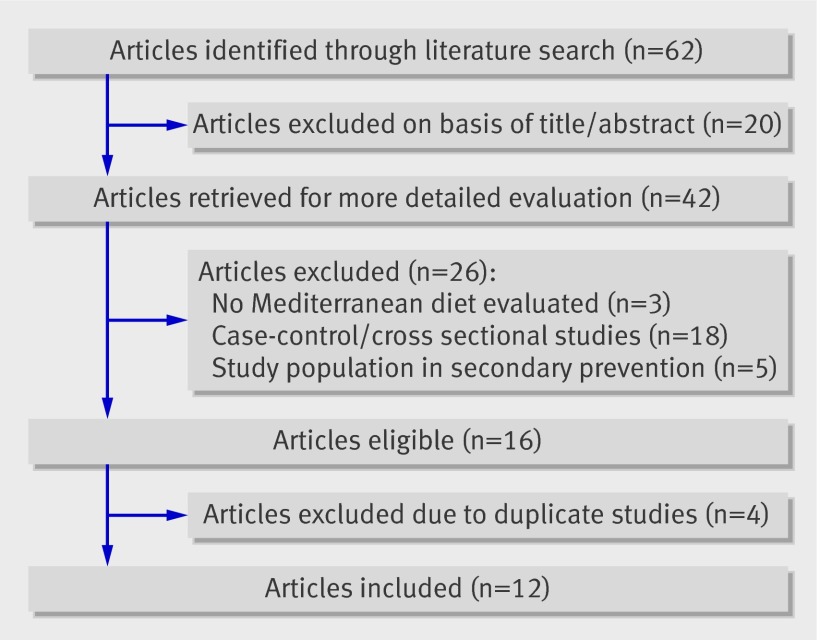
**Fig 1** Process of study selection

### Data extraction

We extracted the following baseline characteristics from the original reports by using a standardised data extraction form and included them in the meta-analysis: lead author, year of publication, cohort name, country of origin of the cohort, sample size of the cohort and number of outcomes, duration of follow-up, age at entry, sex, outcome, components of the score for adherence to a Mediterranean diet, and variables that entered into the multivariable model as potential confounders (table 1). Two investigators (FS and FC) collected the data, and disagreements were solved by consensus and by the opinion of a third author (AC), if necessary. Outcomes of interest were overall mortality, mortality from cardiovascular diseases, incidence of or mortality from cancer, as well as occurrence of Parkinson’s disease and Alzheimer’s disease.

**Table 1 tbl1:** Study characteristics

Author, year	Country	No of outcomes/No in cohort	Outcome	Follow-up (years)	Age at entry (years)	Sex	Components of score	Adjustment
Trichopoulou et al, 1995^w1^	Greece	53/182	Overall mortality	4-5	>70 (mean 75.4)	M/F	1. High legumes; 2. High cereals; 3. High fruits; 4. High vegetables; 5. High MUFA:SFA ratio; 6. Moderate alcohol; 7. Low meat and meat products; 8. Low milk and dairy products	Age, sex, smoking habit, total diet score
Kouris-Blazos et al, 1999^w2^	Australia	36/330	Overall mortality	4-6	≥70	M/F	1. High legumes; 2. High cereals 3. High fruits; 4. High vegetables; 5. High MUFA:SFA ratio; 6. Moderate alcohol; 7. Low meat and meat products; 8. Low milk and dairy products	Age, sex, smoking habit, ethnic origin
Lasheras et al, 2000^w3^	Spain	96/161	Overall mortality	9.5	65-80	M/F	1. High legumes; 2. High cereals; 3. High fruits; 4. High vegetables; 5. High MUFA:SFA ratio; 6. Moderate alcohol; 7. Low meat and meat products; 8. Low milk and dairy products	Age, sex, total diet score, albumin, dieting in response to chronic conditions, BMI, self assessment of health, physical activity
Trichopoulou et al, 2003^w4^ (EPIC)	Greece	275/22 043; 54/22 04	Overall mortality; CHD mortality	3.7	20-86	M/F	1. High legumes; 2. High cereals; 3. High fruits/nuts; 4. High vegetables; 5. High fish; 6. High MUFA:SFA ratio; 7. Moderate alcohol; 8. Low meat and poultry; 9. Low dairy products	Age, sex, smoking habit, years of education, BMI, waist to hip ratio, energy expenditure score, energy intake, consumption of potatoes and eggs
Knoops et al, 2004^w5^ (HALE project: SENECA and FINE)	Belgium, Denmark, Finland, France, Greece, Hungary, Italy, Netherlands, Portugal, Spain, Switzerland	935/2339; 122/2152; 371/2152; 233/2152	Overall mortality; CHD mortality; CVD mortality; cancer mortality	10	70-90	M/F	1. High legumes/nuts/seeds; 2. High cereals; 3. High fruits; 4. High vegetables/potatoes; 5. High fish; 6. High MUFA:SFA ratio; 7. Low meat and meat products; 8. Low dairy products	Age, sex, smoking habit, physical activity, BMI, dietary habits, alcohol, years of education, study population
Trichopoulou et al, 2005^w6^ (EPIC-elderly)	Denmark, France, Germany, Greece, Italy, Netherlands, Norway, Spain, Sweden, UK	3810/67 228 (after exclusion of Greek patients)	Overall mortality	7.4	>60	M/F	1. High legumes; 2. High cereals; 3. High fruits/nuts; 4. High vegetables; 5. High fish; 6. High MUFA+PUFA:SFA ratio; 7. Moderate alcohol; 8. Low meat and poultry; 9. Low dairy products	Age; sex; smoking habit; diabetes; education; BMI; waist to hip ratio; physical activity; energy; consumption of potatoes, eggs, sugar, and confectionery
Lagiou et al, 2006^w7^	Sweden	572/42 237; 280/42 237	Overall mortality; cancer mortality	12	30-49	F	1. High legumes; 2. High cereals; 3. High fruits/nuts; 4. High vegetables; 5. High fish; 6. High MUFA:SFA ratio; 7. Moderate alcohol; 8. Low meat and meat products; 9. Low dairy products	Age; height; BMI; smoking habit; physical activity; education; energy; consumption of potatoes, eggs, PUFA, sweets, and non-alcoholic beverages
Fung et al, 2006^w8^ (nurses’ health study)	USA	3580/71 058	Breast cancer	18	30-55	F	1. High legumes; 2. High wholegrain products; 3. High fruits; 4. High nuts; 5. High vegetables; 6. High fish; 7. High MUFA:SFA ratio; 8. Moderate alcohol; 9. Low red and processed meats	Age, smoking habit, BMI, multivitamins, energy, physical activity, family history of breast cancer and of benign breast disease, menopause, HRT, weight change since age 18 years
Scarmeas et al, 2006^w9^	USA	85/2258	Alzheimer’s disease	4	Mean 77.2	M/F	1. High legumes; 2. High cereals; 3. High fruits; 4. High vegetables; 5. High fish; 6. High MUFA:SFA ratio; 7. Moderate alcohol; 8. Low meat; 9. Low dairy products	Age, sex, cohort, ethnicity, smoking habit, BMI, education, energy, ApoE genotype, comorbidity index
Gao et al, 2007^w10^ (health professionals and nurses’ health studies)—men	USA	508/49 692	Parkinson’s disease	16	40-75	M	1. High legumes; 2. High wholegrain products; 3. High fruits; 4. High nuts; 5. High vegetables; 6. High fish; 7. High MUFA:SFA ratio; 8. Moderate alcohol; 9. Low red and processed meat	Age, smoking habit, BMI, use of NSAIDs, energy, caffeine intake
Gao et al, 2007^w10^ (health professionals and nurses’ health studies)—women	USA	190/81 676	Parkinson’s disease	16	40-75	F	1. High legumes; 2. High wholegrain products; 3. High fruits; 4. High nuts; 5. High vegetables; 6. High fish; 7. High MUFA:SFA ratio; 8. Moderate alcohol; 9. Low red and processed meat	Age, smoking habit, BMI, use of NSAIDs, energy, caffeine intake
Mitrou et al, 2007^w11^ (National Institutes of Health diet and health study)—men	USA	18 126/214 284; 2425/214 284; 3717/214 284	Overall mortality; CVD deaths; cancer deaths	10	50-71	M	1. High legumes; 2. High wholegrain products; 3. High fruits; 4. High nuts; 5. High vegetables; 6. High fish; 7. High MUFA:SFA ratio; 8. Moderate alcohol; 9. Low red and processed meat	Age, race, smoking habit, energy, BMI, education, physical activity
Mitrou et al, 2007^w11^ (National Institutes of Health diet and health study)—women	USA	9673/166 012; 1026/166 012; 2268/166 012	Overall mortality; CVD deaths; cancer deaths	10	50-71	F	1. High legumes; 2. High wholegrain products; 3. High fruits; 4. High nuts; 5. High vegetables; 6. High fish; 7. High MUFA:SFA ratio; 8. Moderate alcohol; 9. Low red and processed meat	Age, race, smoking habit, energy, BMI, education, physical activity, HRT
Benetou et al, 2008^w12^ (EPIC)	Greece	851/25 623	Incident cancers (excluding non-melanoma skin cancers)	7.9	20-86	M/F	1. High legumes; 2. High cereals; 3. High fruits/nuts; 4. High vegetables; 5. High fish; 6. High MUFA:SFA ratio; 7. Moderate alcohol; 8. Low meat and poultry; 9. Low dairy products	Age; sex; smoking habit; years of education; BMI; height; physical activity; total energy; consumption of potatoes, eggs, confectionery, and non-alcoholic beverages

ApoE=apolipoprotein E; BMI=body mass index; CHD=coronary heart disease; CVD=cardiovascular disease; HRT=hormone replacement therapy; MUFA=mono-unsaturated fatty acids; NSAIDs=non-steroidal anti-inflammatory drugs; PUFA=polyunsaturated fatty acids; SFA=saturated fatty acids.

We assessed the quality of the studies according to the number of participants, the duration of follow-up, and adjustment for potential confounders. We considered studies with a high number of participants; long duration of follow-up; and adjustment for confounders including demographic, anthropometric, and traditional risk factors to be of high quality.

### Definition of adherence to Mediterranean diet

Adherence to a Mediterranean diet was defined through scores that estimated the conformity of the dietary pattern of the studied population with the traditional Mediterranean dietary pattern. Values of zero or one were assigned to each dietary component by using as cut offs the overall sex specific medians among the study participants. Specifically, people whose consumption of components considered to be part of a Mediterranean diet (vegetables, fruits, legumes, cereals, fish, and a moderate intake of red wine during meals) was above the median consumption of the population were assigned a value of one, whereas a value of zero was given to those with consumptions below the median. By contrast, people whose consumption of components presumed not to form part of a Mediterranean diet (red and processed meats, dairy products) was above the median consumption of the population had a value of zero assigned, and the others had a value of one. However, some differences among the studies existed, especially in relation to the food category of vegetables (grouped with potatoes in one study^w5^), meat and meat products (grouped with poultry in some studies^w4 w6^), and nuts and seeds (grouped with fruits in some studies,^w4 w6 w7 w12^ with legumes in one study,^w5^ and considered a group by themselves in some others^w8 w10 w11^), as well as milk and dairy products (not present in some studies^w8 w10 w11^) and fish (present only in more recent studies^w4-w12^). Thus, the total adherence scores (estimated as the sum of the above indicated scores of zero and one) varied from a minimum of 0 points indicating low adherence to a maximum of 7-9 points reflecting high adherence to a Mediterranean diet.

### Statistical analysis

We used RevMan, version 4.2 for Windows by the Cochrane Collaboration to analyse data. We used the results of the original studies from multivariable models with the most complete adjustment for potential confounders; table 1 shows the confounding variables included in this analysis. We used a random effects model that accounts for interstudy variation and provides a more conservative effect than a fixed model. We calculated random summary relative risks with 95% confidence intervals by using an inverse variance method. 

We grouped the studies according to the different clinical outcomes (mortality from all causes, mortality from cardiovascular diseases, incidence of or mortality from cancer, and incidence of Parkinson’s disease and Alzheimer’s disease). We assessed the potential sources of heterogeneity by using the standard χ^2^ test. In addition, we used the I^2^ statistic to investigate heterogeneity by examining the extent of inconsistency across the study results. To examine the potential source of heterogeneity across studies evaluating overall mortality, we did sensitivity analyses according to some characteristics of the studies—sex (male, female), country of origin (European countries, United States, other countries), follow-up time (below or above the median follow-up time of the studies: 8 years), and the quality of the studies (low, high). To assess the presence of publication bias, we computed the “failsafe N” for each of the main outcomes; this value is an estimate of the number of studies with null results that would need to be added to the meta-analysis to reduce the overall observed significant result to non-significance.

## Results

### Characteristics of study cohorts

Sample sizes varied between 161 and 214 284, with a follow-up time ranging from 3.7 to 18 years. Outcomes of interest were overall mortality, cardiovascular mortality, incidence of or mortality from neoplastic disease, and incidence of Parkinson’s disease and Alzheimer’s disease. Only six out of 12 studies were done in Mediterranean populations.^w1 w3-w6 w12^ The remaining cohorts comprised US populations,^w8-w11^ northern Europeans,^w5-w7^ and a cohort of Europeans living in Australia.^w3^ The total number of subjects in the included studies was 1 574 299.

### Main outcomes

According to the different clinical outcomes, overall mortality was evaluated in eight cohorts (nine studies) for a total of 514 816 subjects and 33 576 deaths, cardiovascular mortality in three cohorts (four studies) including a total of 404 491 subjects and 3876 fatal events, cancer incidence/mortality in five cohorts (six studies) comprising 521 366 subjects and 10 929 events, and incidence of Parkinson’s disease and Alzheimer’s disease in two cohorts (three studies) for a total of 133 626 subjects and 783 cases.

Figure 2 shows the cumulative analysis for studies that analysed overall mortality as the primary clinical outcome. Using a random effects model, we found that a two point increase in score for adherence to a Mediterranean diet was significantly associated with a reduced risk of mortality from any cause (relative risk 0.91, 95% confidence interval 0.89 to 0.94; P<0.0001). Significant heterogeneity was present among the studies (I_2_=48.8%; P=0.05). However, after exclusion of the paper by Trichopoulou et al 2003 that analysed the same cohort as Trichopoulou et al 2005,^w4 w6^ the significant association with overall mortality remained (relative risk 0.92, 0.91 to 0.94; P<0.0001), showing no significant heterogeneity (I_2_=18.3%; P=0.3).

**Figure fig2:**
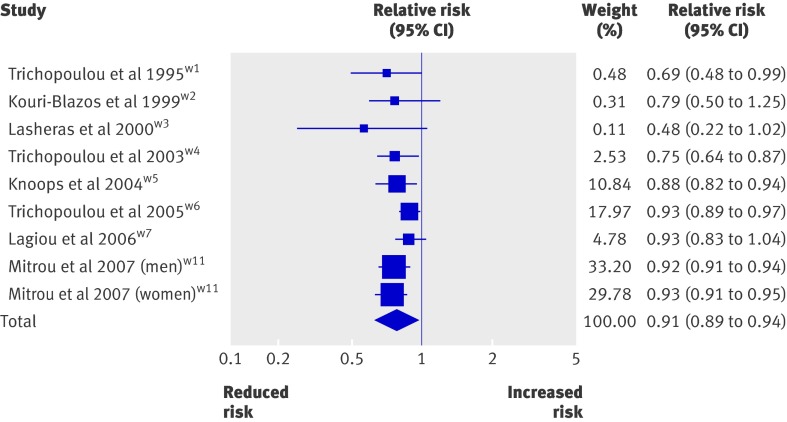
**Fig 2** Risk of all cause mortality associated with two point increase in adherence score for Mediterranean diet. Squares represent effect size; extended lines show 95% confidence intervals; diamond represents total effect size

Similarly figure 3 shows that a greater adherence to a Mediterranean diet significantly reduced the risk of mortality from cardiovascular diseases (relative risk 0.91, 0.87 to 0.95; P<0.0001) with non-significant heterogeneity (I_2_=32.6%; P=0.2). Furthermore, greater adherence to a Mediterranean diet significantly reduced the occurrence of and mortality from neoplasm (relative risk 0.94, 0.92 to 0.96; P<0.0001) (I_2_=0%; P=0.5) (fig 4). Finally, the overall analysis showed a significant reduction in incidence of Parkinson’s disease and Alzheimer’s disease associated with a higher score of adherence to a Mediterranean diet (relative risk 0.87, 0.80 to 0.96; P=0.004), with no heterogeneity among the studies (I_2_=0%; P=0.5) (fig 5).

**Figure fig3:**
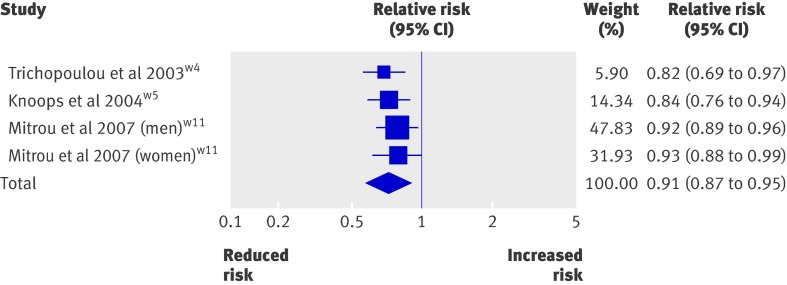
**Fig 3** Risk of mortality from cardiovascular diseases associated with two point increase in adherence score for Mediterranean diet. Squares represent effect size; extended lines show 95% confidence intervals; diamond represents total effect size

**Figure fig4:**
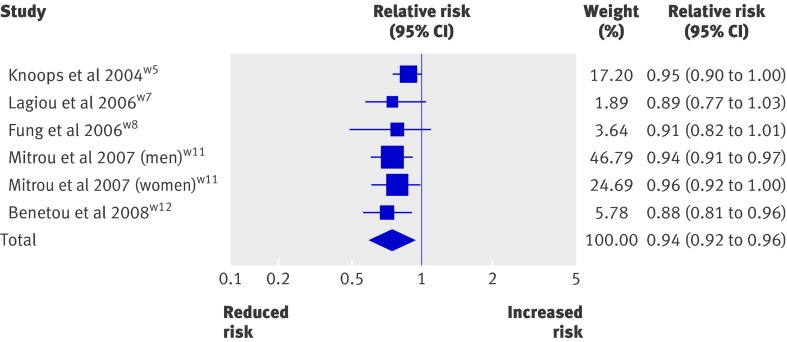
**Fig 4** Risk of occurrence of or mortality from cancer associated with two point increase in adherence score for Mediterranean diet. Squares represent effect size; extended lines show 95% confidence intervals; diamond represents total effect size

**Figure fig5:**
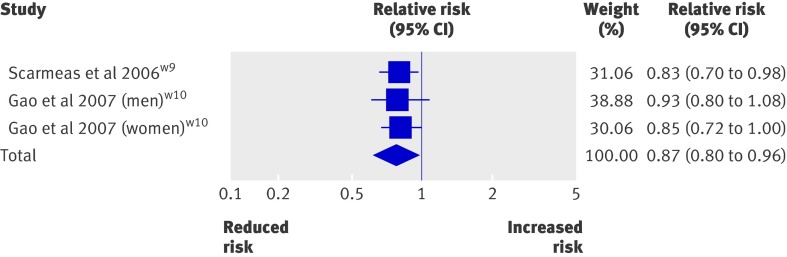
**Fig 5** Risk of Parkinson’s disease and Alzheimer’s disease associated with two point increase in adherence score for Mediterranean diet. Squares represent effect size; extended lines show 95% confidence intervals; diamond represents total effect size

### Sensitivity analyses

Studies included in this meta-analysis varied in some characteristics. Because such heterogeneity of studies is likely to produce heterogeneity of effect sizes across studies, we did some sensitivity analyses. Table 2 shows the different subgroup analyses on studies evaluating overall mortality as clinical outcome. These analyses showed no significant influence of any variable (country of origin of the study, sex, follow-up time, quality of the studies) on the overall results of the meta-analysis.

**Table 2 tbl2:** Sensitivity analysis

Variable (No of studies)	Relative risk (95% CI)
Sex:	
Male (7)	0.89 (0.85 to 0.94)
Female (8)	0.90 (0.86 to 0.94)
Country of origin:	
Europe (6)	0.87 (0.81 to 0.94)
United States/Australia (3)	0.93 (0.91 to 0.94)
Follow-up time:	
<8 years (4)	0.82 (0.69 to 0.97)
>8 years (5)	0.92 (0.91 to 0.94)
Study quality:	
Low (3)	0.89 (0.83 to 0.99)
High (6)	0.92 (0.90 to 0.94)

### Publication bias

To assess the presence of publication bias, we computed the failsafe N for each of the main outcomes. Each failsafe N (580 for studies evaluating overall mortality as main outcome, 68 for studies with cardiovascular mortality as main outcome, 72 for incidence of or mortality from cancer, and 43 for incidence of degenerative diseases) far exceeded Rosenthal’s recommendation (failsafe N>5k+10, where k is the number of studies included in the analysis) for a robust effect of the overall analysis.

## Discussion

This meta-analysis shows, in an overall analysis comprising more than 1.5 million healthy subjects and 40 000 fatal and non-fatal events, that greater adherence to a Mediterranean diet is significantly associated with a reduced risk of overall mortality, cardiovascular mortality, cancer incidence and mortality, and incidence of Parkinson’s disease and Alzheimer’s disease. The cumulative analysis of 12 cohort studies shows that a two point increase in the score for adherence to a Mediterranean diet determines a 9% reduction in overall mortality, a 9% reduction in mortality from cardiovascular diseases, a 6% reduction in incidence of or mortality from neoplasm, and a 13% reduction in incidence of Parkinson’s disease and Alzheimer’s disease. To the best of our knowledge, this is the first report that has systematically assessed, through meta-analysis, the possible association between adherence to a Mediterranean diet, mortality, and the occurrence of chronic diseases in the general population.

### Diet and disease

The effect of diet on human health has been amply reported in many epidemiological, population based, and randomised clinical trials, providing evidence that a dietary pattern rich in some beneficial food groups such as fruit, vegetables, whole grains, and fish can reduce the incidence of cardiovascular and neoplastic diseases.^7^ However, until now, the vast majority of studies followed the approach of assessing single nutrients or food groups in relation to the occurrence of disease.^4^ ^8^ ^9^ This approach seems to have several conceptual and methodological limitations, because food components of diet present synergistic and antagonist interactions and because people eat a complex of nutrients.^5^ Therefore, over the past few years, researchers have shifted their attention from the evaluation of single nutrients to the analysis of dietary pattern as a whole.^6^ ^w1-w10^ As a result, an increasing number of studies have been done by summing foods considered to be important for health to provide an overall measure of dietary quality—that is, a quality diet score.^6^

In this context, a prominent position has been occupied by studies evaluating adherence to a Mediterranean diet, because of its well known and evidence based beneficial effects on human health. Indeed, since the early 1970s many investigators have reported the beneficial role of the Mediterranean diet, as originally reported by Keys in the pioneering seven countries study.^3^ A diet rich in fruits, vegetables, legumes, and cereals, with olive oil as the only source of fat, moderate consumption of red wine especially during meals, and low consumption of red meat has been shown to be beneficial for all cause and cardiovascular mortality, lipid metabolism, blood pressure, and several different disease states such as endothelial dysfunction and overweight.^7^

### Practical implications

In this study we aimed to systematically analyse all the prospective cohort studies that evaluated the effect of a computational score estimating adherence to a Mediterranean diet on health status. From the overall analysis of 11 cohort studies, of which eight assessed the risk of overall mortality, four assessed cardiovascular mortality, six assessed incidence of or mortality from neoplasm, and three assessed incidence of Parkinson’s disease and Alzheimer’s disease, we report a significant reduction in risk of all the main clinical outcomes with an increasing score for adherence to a Mediterranean diet. This observation seems to show that a score based on a theoretically defined Mediterranean diet is an effective preventive tool for measuring the risk of mortality and morbidity in the general population.

A Mediterranean diet has been shown to have a beneficial effect on the occurrence of diseases in industrialised and non-industrialised countries. All the major scientific associations, in fact, strongly encourage people to consume a Mediterranean-like dietary pattern to reduce their risk of disease.^10^ ^11^ ^12^ Unfortunately, despite this worldwide promotion of the Mediterranean diet, a progressive shift to a non-Mediterranean dietary pattern, even in countries bordering the Mediterranean sea, has progressively developed.^13^ It thus seems urgent to identify an effective preventive strategy to decrease the risk burden related to dietary habits in the general population; the use of such a tool could be important in increasing the implementation of dietary guidelines.

### Limitations

Some limitations of this study can be identified. The Mediterranean diet is not a homogeneous pattern of eating, and heterogeneity on the score items exists. How to group some food categories such as legumes, nuts, and milk and dairy products; the real importance of different types of meat; and the establishment of the moderate amount of alcohol intake are still matters of dispute among researchers and can differ among the selected studies. None the less, the key characteristics of a Mediterranean diet were present in all the studies, and the overall analysis seemed not to be significantly influenced by these differences. In addition, the use of a score for estimating a dietary pattern is limited by subjectivity, conditioned by the available data and the main objectives of the study, and so possibly determining a great variability in the interpretation of the results.

Finally, a further limitation exists in the different adjustment for potential confounders seen among the included studies. This difference could have determined a residual confounding within the studies, especially for the non-Mediterranean cohorts. However, the sensitivity analysis according to the quality of the studies, which also included the presence or not of adjustment factors, showed no significant influence of residual confounding on the overall findings of our meta-analysis.

### Conclusions

This meta-analysis shows that adherence to a Mediterranean diet can significantly decrease the risk of overall mortality, mortality from cardiovascular diseases, incidence of or mortality from cancer, and incidence of Parkinson’s disease and Alzheimer’s disease. These results seem to be clinically relevant in terms of public health, particularly for reducing the risk of premature death in the general population, and are strictly concordant with current guidelines and recommendations from all the major scientific associations that strongly encourage a Mediterranean-like dietary pattern for primary and secondary prevention of major chronic diseases.

**What is already known on this topic**

The Mediterranean diet is a well known model of diet for primary and secondary prevention of major chronic diseases
An adherence score can be used to assess the adherence of a specific population to the rules of a traditional Mediterranean diet

**What this study adds**

Greater adherence to a Mediterranean diet confers a significant protection for overall mortality, as well as cardiovascular disease mortality and incidence of cancer and degenerative diseases
The adherence score based on a theoretically defined Mediterranean diet could be an effective preventive tool for reducing the risk of mortality and morbidity in the general population

## Notes

Ethical approval: Not needed.

Provenance and peer review: Not commissioned; externally peer reviewed.

## Notes

**Cite this as:** *BMJ* 2008;337:a1344
